# Dedifferentiated liposarcoma of the extremities: a Korean multi-center study of 107 cases

**DOI:** 10.1186/s12885-024-13021-y

**Published:** 2024-10-10

**Authors:** Jay Hoon Park, Sung Eun Kim, Wanlim Kim, Youngsung Kim, June Hyuk Kim, Sung Wook Seo, Han-Soo Kim, Shinn Kim, Ilkyu Han

**Affiliations:** 1 Department of Orthopaedic Surgery, Seoul National University Hospital, Seoul, Korea; 2 Department of Translational Medicine, Seoul National University College of Medicine, Seoul, Korea; 3 Department of Orthopaedic Surgery, Seoul National University College of Medicine, Seoul, Korea; 4 Department of Orthopaedic Surgery, Asan Medical Center, University of Ulsan College of Medicine, Seoul, Korea; 5 Department of Orthopaedic Surgery, Seoul National University Bundang Hospital, Seongnam, Korea; 6 Orthopaedic Oncology Clinic, National Cancer Center, Goyang, Gyeonggi, Korea; 7 Department of Orthopaedic Surgery, Samsung Medical Center, Seoul, Korea; 8 Musculoskeletal Tumor Center, Seoul National University Cancer Hospital, 101 Daehak-Ro Jongno-Gu, Seoul, 03080 Korea

**Keywords:** Dedifferentiated liposarcoma, Extremities, Soft tissue sarcoma, Multi-center, Oncologic outcome, Local recurrence, Metastasis, Disease specific survival

## Abstract

**Background:**

Dedifferentiated liposarcoma of the extremities (DDL-E) is rare in comparison to that of the retroperitoneum. Its clinical features and surgical principle for resection margins at the dedifferentiated and the well-differentiated components are yet to be elucidated.

**Methods:**

This retrospective multi-center study examined patients diagnosed with DDL-E from August 2004 to May 2023 at 5 sarcoma centers. Clinical features, oncologic outcomes, and prognostic factors were analyzed.

**Results:**

A total of 107 patients were reviewed. The 5-year local recurrence free survival (LRFS), metastasis-free survival (MFS) and disease specific survival (DSS) were 84.7%, 78.6%, and 87.8%, respectively. Other primary malignancies and extrapulmonary metastasis were observed in 27 and 4 patients, respectively. The independent risk factor for local recurrence was R1/2 margin at the dedifferentiated component of the tumor. Metastasis was associated with tumor size in univariate analysis. The independent risk factor for DSS was tumor grade. Previous unplanned excision, de novo presentation, tumor depth, absence of the well-differentiated component, infiltrative border, R1/2 margin at the well-differentiated component were not associated with oncologic outcomes.

**Conclusions:**

This is the largest study examining DDL-E to-date. Localized DDL-E has low potential for metastasis and carries an excellent prognosis. Other primary malignancy and extrapulmonary metastasis are more frequent in DDL-E, thus close monitoring of other sites during follow-up is recommended. While wide resection margin is the standard surgical approach for DDL-E, further investigation into moderated wide resection margin at the well-differentiated component is warranted.

## Introduction

The definition of dedifferentiated liposarcoma (DDL) has evolved since Evans et al. [1] first described the disease entity in 1979 as biphasic sarcoma, in which well-differentiated liposarcoma is accompanied by high-grade dedifferentiated component. Henricks et al. [2] expanded the diagnostic definition to include DDL with low-grade dedifferentiated component, which demonstrated greater capacity to metastasize and cause death, unlike Well-differentiated Liposarcoma (WDL). The current definition of DDL not only includes WDL with high or low grade dedifferentiated component, but also non-lipogenic spindle cell or pleomorphic sarcoma with identifiable Mouse Double Minute 2 (MDM2) amplification even in the absence of WDL component [3, 4].

Prevalent locations for DDL are retroperitoneum (RP), extremities, trunk wall, and inguinal lesion. Dedifferentiated liposarcoma of the RP (DDL-RP) occupy up to 90% of the DDL [4], and has shown distinctively poorer prognosis compared to DDL of other sites, possibly due to its occult location [2]. Dedifferentiated liposarcoma of the extremities (DDL-E) are much rarer. Most previous studies, which examined the clinical characteristics and prognosis of DDL-E, had small cohort of less than 30 [2, 5–10]. Tseng et al. and Morii et al. reported on DDL-E and the trunk wall with greater patient size of 58 and 132, respectively [11, 12]. Amongst the few studies, reported clinical characteristics, oncologic outcomes, and prognostic factors vary. Nakata et al. [10] noted high likelihood of DDL-E being associated with secondary malignancies, which was not examined in other studies. Predilection for extra-pulmonary metastasis were noted, yet the favored locations varied [6, 10, 12]. Prognostic factors for local recurrence, metastasis, and disease specific survival also differed, while tumor grade and the size of dedifferentiated component were commonly noted.

Our study aimed to evaluate 1) clinical characteristics, 2) oncologic outcomes (local recurrence, metastasis, and disease specific survival), and 3) prognostic factors of DDL-E.

## Methods

### Study design

This retrospective, multi-center study examined electronic medical records of patients diagnosed with DDL-E from August 2004 to May 2023 at 5 sarcoma centers in South Korea. Inclusion criteria were pathologic diagnosis of DDL-E. DDL-E in inguinal lesion was included if the location was extra-abdominal. Exclusion criteria were DDL arising from RP, trunk wall, and scrotum. Other exclusion criteria were metastasis or recurrence at initial presentation, no surgical treatment, and follow up loss with follow up less than 2 years.

The diagnosis of DDL was made by pathologists based on the identification of transition from WDL to non-lipogenic sarcoma, detection of non-lipogenic sarcoma with WDL component, or, in the absence of WDL component, non-lipogenic sarcoma with the amplification of MDM2 in immunohistochemistry or Fluorescence in situ hybridization (FISH).

Our study was approved by the Institutional Review Boards at each center.

### Treatment

All the patients underwent surgical treatment of DDL-E. No neoadjuvant chemotherapy was performed. Adjuvant chemotherapy was administered on high grade, R1/2 pathologic margin or metastatic cases. The first line regimen included anthracycline or in combination with ifosfamide. The second line regimen included ifosfamide, carboplatin, etoposide (ICE regimen), or cyclophosphamide, vincristine, adriamycin, dacarbazine (CYVADIC regimen). Neo or adjuvant radiation therapy was carried out for large size, high grade, or R1/2 pathologic margin cases with averagae dosage around 60 grey (Gy).

### Evaluation

Following variables were extracted from the electronic medical records of the participants: age, sex, unplanned excision at initial presentation, tumor type (de novo versues secondarily differentiated from previous lipoma or WDL), location, presence of WDL in Magnetic Resonance Imaging (MRI), tumor depth (subcutaneous versus deep), tumor size, metastasis site, tumor grade, pathologic border type (expansile versus infiltrative), pathologic margin at the well-differentiated and dedifferentiated component (R0 versus R1/2), MDM2 amplification, (neo)adjuvant radiotherapy, adjuvant chemotherapy, other primary malignancies, local recurrence free survival, metastasis free survival, and disease specific survival.

Tumor type (de novo versus secondary) was determined based on the previous record of lipoma or WDL before the diagnosis of DDL-E at the same location. Tumor size was recorded based on the pathology report. Metatstais was diagnosed based on pathologic confirmation through core needle biopsy or definite increase of the mass size in serial imaging follow up. Tumor grade was assessed using the modified Fédération Nationale des Centres de Lutte Contre le Cancer (FNCLCC) grading system for soft tissue tumor [13, 14]. Pathologic margins were evaluated using the R classification [15]. Measurement bias of the variables was addressed because all the aforementioned variables had observer-independent, objective records.

### Statistical analysis

The starting point of oncologic outcomes was the date of initial surgery. The endpoints were local recurrence, metastasis, and disease specific survival.

Prognostic factors were investigated using uni- and multi-variate analysis. For the univariate anlaysis, Kaplan–Meier curve and log rank test was used. Factors with *p* < 0.1 were included for the multi-variate Cox Proportional Hazard analysis. Continous variables (age and tumor size) were converted to categorical variables for the prognostic factor analysis. Optimal cutoffs for the continuous variables were defined by Youden index using Receiver Operating Characteristic (ROC) curve analysis [16].

Statistical significance was defined as *p* < 0.05. Statistical analysis was performed using IBM SPSS Statistic (v26.0. Armonk, NY: IBM Corp).

## Results

### Clinical characteristics

**Fig. 1:**
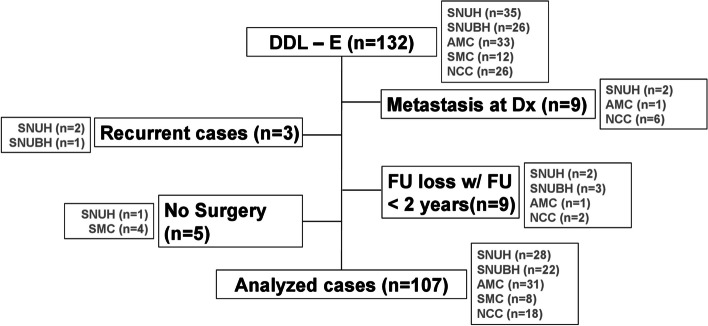
The flow diagram of the enrolled patients. DDL-E, dedifferentiated liposarcoma of the extremities; Dx, diagnosis; FU, follow-up

The study examined 107 patients under the aforementioned criteria (Fig. 1). Clinical characteristics of the patients are summarized in Table 1. There were 70 males and 37 females. Median age was 64 (range 19–88). Ethnicity of all study participants was Korean. At initial visit, 80 presented without previous treatment, and 27 with unplanned excision.

**Table 1:** Baseline characteristics 107 dedifferentiated liposarcoma patients

**Patient Demographics**		Pathologic Margin	
Patients	107	Overall	
Age	64 (19–88)	R0	69.2 (74)
Gender		R1/2	30.8 (33)
Female	34.6 (37)	WD Component	
Male	65.4 (70)	R0	77.4 (41)
Follow Up (months)	36 (15.8–61)	R1/2	22.6 (12)
**Tumor**		DD Component	
Initial Presentation		R0	75.7 (81)
Fresh	74.8 (80)	R1/2	24.3 (26)
Unplanned Excision	25.2 (27)	Grade	64.5 (69)
Tumor Type		1	3.7 (4)
De Novo	88.8 (95)	2	48.6 (52)
Secondary	11.2 (12)	3	36.4 (39)
Size	10.5 (0.5–35)	Not Assessed	11.2 (12)
Depth		Border	
Superficial	19.6 (21)	Expansile	13.1 (14)
Deep	80.4 (86)	Infiltrative	45.8 (49)
Location		Not Assessed	41.1 (44)
Shoulder	12.1 (13)	MDM2	
Upper Arm	6.5 (7)	Positive	64.5 (69)
Elbow	1.9 (2)	Negative	7.5 (8)
Forearm	1.9 (2)	Not Assessed	28.0 (30)
Hand	0.9(1)	Other Primary Malignancy	22.4 (20)
Buttock	6.5 (7)	**Treatment**	
Inguinal	11.2 (12)	Chemotherapy	
Thigh	54.2 (58)	Neoadjuvant	0 (0)
Knee	0.9 (1)	Adjuvant	28.0 (30)
Lower Leg	2.8 (3)	Radiotherapy	
Ankle	0.9 (1)	Neoadjuvant	1.9 (2)
		Adjuvant	68.2 (73)

*Abbreviations:* *WD* well-differentiated, *DD* de-differentiated

Data presented as % (n) or median (interquartile range)

Tumor size is in centimeters

A total of 95 (88.8%) cases were de novo DDL, and 12 (11.2%) were secondary DDL. WDL was present in the MRI of 60 (56.1%) patients. Median tumor size was 10.5 cm (range 0.5–35). In terms of tumor depth, 21 (19.6%) tumors were located subcutaneously, and 86 (80.4%) were located deep. The tumor locations were as follows: upper extremity 25 cases (shoulder 13, upper arm 7, elbow 2, forearm 2, hand 1), lower extremity 82 cases (buttock 7, inguinal 12, thigh 58, knee 1, lower leg 3, ankle 1).

Pathologic margin was R1/2 in 33 (30.8%) of cases. The well-differentiated component had R1/2 margin in 12 (22.6%) cases. The dedifferentiated component had R1/2 pathologic margin in 26 (24.3%) cases. Tumor grade was grade 1 in 4 (3.7%) cases, grade 2 in 52 (48.6%) cases, grade 3 in 39 (36.4%) cases. Tumor grade could not be assess in 12 (11.2%) cases: 2 because no residual tumor was present after the unplanned excision, and 10 because the pathology report did not include tumor grade data. Tumor border was expansile in 14 (13.1%) cases, infiltrative in 49 (45.8%) cases, and not reported in 44 (41.1%) cases. MDM was amplified in in 69 (64.5%) cases, negative in 8 (7.5%) cases, and not assessed in 30 (28.0%) cases.

Perioperative radiotherapy was performed in 75 (70.1%) cases (neoadjuvant 2, adjuvant 73). Median radiation dosage was 60 (range 45–72). Chemotherapy was performed in 30 (28.0%) cases (neoadjuvant 0, adjuvant 30).

Other primary malignancies were present in 24 (22.4%) cases. The type of cancer for other primary malignancy varied widely from colon, liver, breast, lung, prostate, kidney, endometrial cancer to lymphoma, without having a predilection for a specific cancer type.

### Oncologic outcomes

**Fig. 2:**
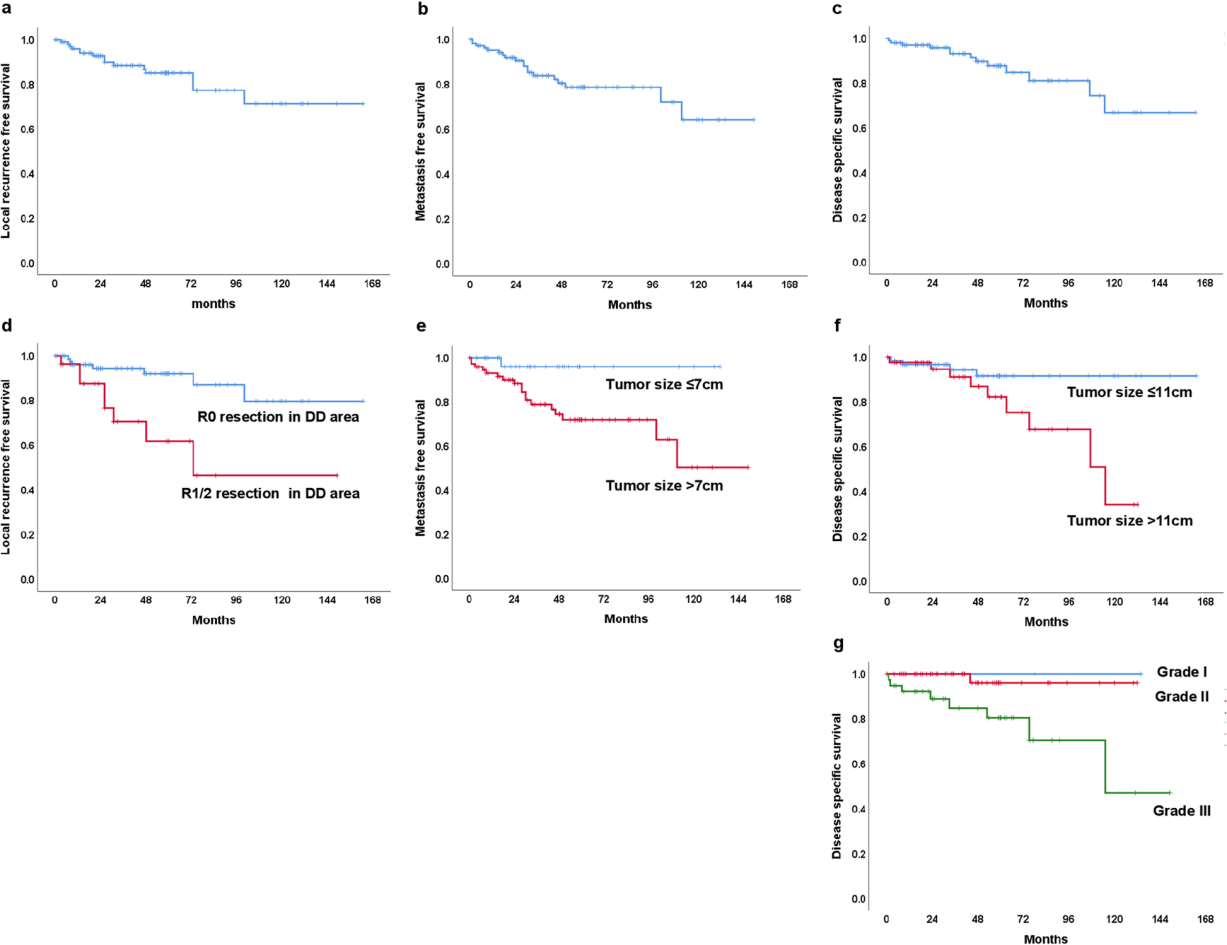
**a** Kaplan–Meier curve for the local recurrence-free survival. **b** Kaplan–Meier curve for the metastasis free survival. **c** Kaplan–Meier curve for the disease specific survival. **d**-**g** Kaplan–Meier curve for the significant prognostic factors. **d** Pathologic margin at the dedifferentiated area as significant risk for local recurrence. **e** Tumor size as significant risk for metastasis. **f** Tumor size and **g** tumor grade as significant risk for disease specific survival

Median follow up was 36 (interquartile range 15.8–61) months. Local recurrence, metastasis, and death were observed in 15 (14.0%), 19 (17.7%), and 13 (12.1%)of the participants, respectively (Fig 2a-c). Extrapulmonary metastases were observed in 4 of 19 cases: The locations were calf in one case, pancreas in one case, T3 spinal vertebra and temporal bone in one case, and pleura in one case. Five-year local recurrence, metastasis, and disease specific survival were 84.7%, 78.6%, and 87.8% respectively (Fig. 2a-c).

### Prognostic factor

The cutoff values of continuous variables (age and tumor size) for survival analysis are displayed in Table 2. In terms of local recurrence, R1/2 pathologic margin in dedifferentiated component showed significant association (*p* = 0.002) in the univariate analysis (Table 3, Fig. 2d). R1/2 pathologic margin in dedifferentiated component was also an independence risk factor (*p* = 0.008, Hazard Ratio [HR] 4.23, 95% Confidence Interval [CI] 1.45–12.26) associated with local recurrence. In terms of metastasis, tumor size showed significant association (*p* = 0.014) in the univariate analysis (Table 4, Fig. 2e). In terms of disease specific survival, tumor size (*p* = 0.024) and tumor grade (*p* = 0.017) showed significant association in the univariate analysis (Table 5, Fig. 2f-g). Tumor grade (*p* = 0.048, HR 8.48, 95% CI 1.02–69.0) was an independence risk factor associated with disease specific survival.

**Table 2:** Result of cutoff values for continuous variables for oncologic outcomes using receiver operatic curve analysis

	**Local Recurrence**		**Metastasis**		**Disease Specific Survival**	
	**AUC**	**Cutoff**	**AUC**	**Cutoff**	**AUC**	**Cutoff**
**Age**	0.55	54	0.58	54	0.48	54
**Tumor Size**	0.58	8.65	0.65	7.15	0.63	11.35

Tumor size is in centimeters

**Table 3:** Prognostic factors for local recurrence

Variable		5-year survival (%)	P (univariate)	P (multivariate)	Hazard ratio	95% CI
	Less than 53	88.1	0.761			
Age	54 and older	83.2				
Sex	Male	80.8	0.209			
	Female	92.8				
Initial Presentation	Fresh	85	0.901			
	Unplanned OP	85.7				
Tumor Type	De Novo	88.8	0.069	(Reference)		
	Secondary	60		0.088	2.82	0.86–9.28
Depth	Subcutaneous	95	0.209			
	Deep	82.6				
Tumor size	less than 8.65 cm	91.6	0.089	(Reference)		
	Over 8.65 cm	78.9		0.669	1.26	0.43–3.70
MRI Classification	WD component ( +)	83.6	0.685			
	WD component (-)	86.4				
FNCLCC	Grade 1	75	0.887			
	Grade 2	85.8				
	Grade 3	87				
Border	Expansile	83.3	0.202			
	Infiltrative	88.9				
Pathologic margin	R0	91.3	0.028			
	R1/2	69.8				
Pathologic margin (WD)	R0	85.1	0.808			
	R1/2	77.9				
Pathologic margin (DD)	R0	92	0.002	(Reference)		
	R1/2	61.8		0.008	4.23	1.46–12.26
Periop RT	Yes	84.7	0.999			
	No	86.1				

*Abbreviations: CI* Confidence interval, *WD* Well-differentiated, *FNCLCC* Fédération Nationale des Centres de Lutte Contre le Cancer, *DD* De-differentiated

**Table 4:** Prognostic factors for metastasis

Variable		Five-year survival (%)	P (univariate)
Age	Less than 54	82.8	0.318
	54 and older	77.4	
Sex	Male	79.6	0.797
	Female	78	
Initial Presentation	Fresh	79.4	0.898
	Unplanned OP	75.5	
Tumor Type	De Novo	78	0.306
	Secondary	85.7	
Depth	Subcutaneous	93.3	0.119
	Deep	75.2	
Tumor size	less than 7.15 cm	96	0.014
	Over 7.15 cm	71.9	
MRI Classification	WD component ( +)	78.2	0.827
	WD component (-)	79.2	
FNCLCC	1	100	0.185
	2	88	
	3	67.3	
Border	Expansile	79.1	0.7
	Infiltrative	82	
Pathologic margin	R0	79.9	0.775
	R1/2	74.5	
Pathologic margin (WD)	R0	81.8	0.198
	R1/2	56.8	
Pathologic margin (DD)	R0	80.3	0.699
	R1/2	71.3	
**Periop RT**	**Yes**	**73.6**	**0.476**
	**No**	**93.4**	

*Abbreviations: CI* Confidence interval, *WD* Well-differentiated, *FNCLCC* Fédération Nationale des Centres de Lutte Contre le Cancer, *DD* De-differentiated

Multivariate analysis was not performed, since only ‘tumor size’ had *p* < 0.1

**Table 5:** Prognostic factors for disease specific survival

Variable		Five-year survival (%)	P(univariate)	P (multivariate)	Hazard ratio	95% CI
Age	Less than 54	89.3	0.528			
	54 and older	87.6				
Sex	Male	87.9	0.717			
	Female	87.3				
Initial Presentation	Fresh	91.4	0.336			
	Unplanned OP	74.1				
Tumor Type	De Novo	88.4	0.55			
	Secondary	85.7				
Depth	Subcutaneous	87.7	0.756			
	Deep	87.9				
Tumor size	less than 11.35 cm	91.6	0.024	(Reference)		
	Over 11.35 cm	82.1		0.142	3.34	0.67–16.68
MRI Classification	WD component ( +)	87.7	0.785			
	WD component (-)	89				
FNCLCC	1	100	0.017	(Reference)		
	2	96.2		(Reference)		
	3	79.6		0.048	8.38	1.02–69.0
Border	Expansile	84.6	0.21			
	Infiltrative	92.8				
Pathologic margin	R0	92.3	0.262			
	R1/2	76.1				
Pathologic margin (WD)	R0	90.5	0.137			
	R1/2	62.5				
Pathologic margin (DD)	R0	92.9	0.08	(Reference)		
	R1/2	69.4		0.376	1.88	0.47–7.59
Periop RT	Yes	87.9	0.866			
	No	88.1				

*Abbreviations: CI* Confidence interval, *WD* Well-differentiated, *FNCLCC* Fédération Nationale des Centres de Lutte Contre le Cancer; *DD* De-differentiated

## Discussion

### Clinical characteristics

This is the largest restrospective cohort study investigating dedifferentiated liposarcoma of the extremities to-date (Table 6). Previously reported clinical characteristics of DDL-E such as middle-aged adult, predilection for male, and prevalent location of thigh were validated in this study [2, 5–8, 10–12, 17]. Interestingly, the 2020 World Health Organization (WHO) Classification of Soft Tissue Tumours [4] stated that DDL has peak incidence between forth and fifth decades of life and no gender predilection. Nevertheless, previous literature on DDL-E, including this study, had median or mean participant age around the seventh decade, and clear predilection for male patients [2, 5–8, 10–12, 17]. Frequent age group and gender diagnosed with DDL-E and DDL-RP do not seems to differ [2, 18, 19]. The proportion of de novo versus secondary DDL in this study was nearly identical to the previously reported values [4, 12, 20].

**Table 6:** Oncologic outcomes of dedifferentiated liposarcoma of the extremities in the present and previous studies

Publication	Author	Study Size	Local Recurrence		Metastasis		Lethal Event	
			Rate	Prognostic factor	Rate	Prognostic factor	Rate	Prognostic factor
1993	Kransdorf	4	0/4 (0%)		1/4 (25%)		0/4 (0%)	
1994	McCormick	7	2/7 (29%)		2/7 (29%)		1/7 (14%)	
1997	Henricks	27	9/27 (33.3%)		4/27 (14.8%)		3/27 (11.1%)	
2006	Hoshi	3	0/3 (0%)		0/3 (0%)		0/3 (0%)	
2011	Okada	15	1/15 (6.6%)		4/15 (26.6%)		4/15 (26.6%)	DD area size^**d**^, MIB-1 index^**d**^, Lung metastasis^**d**^
2016	Kito	7	0/7 (0%)		2/7 (28.6%)		2/7 (28.6%)	
2020	Nakata	15	3/15 (20%)	Tumor size^**d**^	3/15 (20%)		1/15 (6.6%)	
2021	Barlett^a, b^		32.60%		18.00%		12.60%	
2021	Tseng^a^	58	8/58 (14.2%)	Tumor grade^**d**^	5/58 (8.6%)		6/58 (10.7%)	Tumor grade^**d**^, DD area size^**d**^
2023	Morii^a^	132^c^	34/132 (25.8%)	Percentage of DD area, Marginal excision, DD Margin	32/132 (24.2%)	DD area size, Tumor grade, Marginal excision	18/132 (13.6%)	DD area size, Lung Metastasis
	Present Study	107	15/107 (14%)	DD margin	19/107 (17.7%)	Tumor size^**d**^	13/107 (12.1%)	Tumor grade

Oncologic outcomes are indicated by event per total cases (percentage)

*DD* Dedifferentiated

^a^Dedifferentiated liposarcoma of the extremities and the trunk wall are reported

^b^Cumulative incidence at 10 years is reported

^c^Of 132, 92 were located in the extremities and 40 in the trunk wall

^d^Prognostic factors show significance of *p* < 0.05 in the univariate analysis

Previous literature on increased likelihood of other primary malignancies in DDL-E was confirmed in our study (22.4%). Nakata et al. [10] reported increased likelihood of other primary malignancies in DDL-E (5 of 15 [33.3%]). Jung et al [21] also described increased risk of additional primary malignancy in WDL and DDL (26 of 312 [8.3%]), although 15 DDL cases of the 26 were all located in the RP. Considering the reported frequency of multiple primary malignancies for all tumors (2–17%) [22], DDL-E appears to have higher risk for other primary malignancies. The etiology remains unclear as 12q13–15 amplification, a genetic hallmark of DDL, is yet to show association with other cancer syndromes [10, 21]. Further investigation into the genetic predisposition of patients with DDL-E is warranted. Although this study did not include comprehensive genetic testing, future studies could focus on identifying genetic mutations that may contribute to both DDL-E and other primary malignancies. In addition, close examination and monitoring of DDL-E patients for other primary malignancy is recommended.

### Oncologic outcomes

Five-year local recurrence, metastasis, and disease specific survival were 84.7%, 78.6%, and 87.8% respectively. The oncologic outcomes in this study were comparable to previous listerature on DDL-E (Table 6). Localized DDL-E demonstrates low potential for metastasis and an excellent prognosis compared to DDL-RP or localized soft tissue sarcoma as a whole [2, 23, 24]. The significantly better prognosis of DDL-E compared to DDL-RP is likely attributable to several factors. Anatomically, DDL-E lesions in the extremities are easier to detect and resect with clear margins, while DDL-RP lesions often present in late stages due to their deep location. Additionally, retroperitoneal tumors tend to have higher local recurrence rates due to challenges in achieving clear resection margins.

Local recurrence in this study was notably lower than that in the study by Morii et al., the only other large study on DDL-E to-date (15 out of 107 [14.0%] in this study versus 34 out of 132 [25.8%] in the study by Morii et al. [12]) (Table 6). Clinical features, such as age, gender, ethnic group, tumor size, depth, grade, proportions of adjuvant chemotherapy, and R1/2 margin at the dedifferentiated component (26 of 107 [24.3%] versus 34 of 132 [25.8%] [12]) were comparable between the two studies. However, there was a stark difference in the proportions of patients who received adjuvant radiotherapy (74 of 107 [69.2%] versus 27 of 132 [20.4%]). In this study, 21 of 26 (80.7%) patients, who had R1/2 margin at the dedifferentiated component, received adjuvant radiotherapy. In the study by Morii et al., 3 of 34 (8.8%) patiens, who had R1/2 margin at the dedifferentiated component, received adjuvant radiotherapy. Based on the difference, we assert that adjuvant radiotherapy may play a role in reducing the local recurrence of DDL-E. This analysis is supported by Bartlett et al., who reported that DDL-E patients who receiced radiotherapy had lower 5-yer local recurrenc rate [17]. In addition, Haas et al. affirmed that radiotherapy is associated with local recurrence in DDL-RP in univariate analysis by directly comparing cases of surgery plus radiotherapy and surgery only [25]. Although perioperative radiotherapy was not an independent prognostic factor for local recurrence in both studies, the role of adjuvant radiotherapy in local recurrence cannot be neglected because deliberate selection of patients (large tumor size, high tumor grade, R1/2 pahotlogic margin) to receive adjuvant radiotherapy acts as a confounding variable.

Previously reported high frequency of extrapulmonary metastases in DDL-E by Bartlett et al. (6 of 11 [54.5%]) [17] and Morii et al. (11 of 32 [34.4%]) [12], was verified in this study (4 of 19 [21.1%]). In the case of metastasis to the calf, the location was distant from the primary tumor and occurred after the initial surgery, suggesting a metastasis. However, the possibility of a second primary tumor or direct extension cannot be entirely ruled out. High index of suspicion for potential extrapulmonary metastases or other primary malignancies at locations other than the initial tumor or the lung is recommended during the follow-up of DDL-E patients.

### Prognostic factor

To this point, only a few studies have investigated the prognostic factors for the oncologic outcome of DDL-E [8, 10–12]. Radiologic parameters such as tumor size, dedifferentiated component size and percentage had been examined as prognostic factors. Okada et al. reported that dedifferentiated area size > 8 cm (cm) was associated with disease specific survival [8] (Table 6). Nakata et al. demonstrated that tumor size > 15 cm was associated with local recurrence [10]. Morii et al. indicated that the area of dediffentiated are > 36cm^2^ and > 77cm^2^ were associated with metastasis and disease specif survival, respectively [12]. In this study, tumor size > 7.15 cm was an independent factor for metastasis. Although specific outcomes vary by studies, tumor size has repeatedly demonstrated its role as a prognostic factor for DDL-E. Morii et al. also looked into the percentage of dedifferentiated area within the entire tumor, and illustrated its association with local recurrence [12]. In this study, composition of the tumor was approached as a categorical variable: presence or absence of WDL component within the tumor. Absence of WDL component was not associated with oncologic outcomes in DDL-E.

Tumor grade had been reported as a prognostic factor for disease specific survival in DDL-E, supporting the result of this study [8, 11]. Okada et al. reported that MIB-index, a strong indicator for grading of soft tissue sarcoma, is associated with disease specific survival in DDL-E [8, 26]. Tseng et al. also indicated that tumor grade is associated with disease specific survival in DDL-E [11].

Pathologic margin, specifically margin at the well-differentiated an the dedifferentiated components, as a prognostic factor for local recurrence had been actively investigated. Hoshi et al. was the first to state that wide resection margin at the dedifferentiated component and marginal resection margin at the well-differentiated component suffice for local control [7]. Our study confirmed Morii et al.’s result that R1/2 margin at the differentiated component is an independent factor for local recurrence, while R1/2 margin at the well-differentiated component is not [12]. Morii et al. asserted that although R1/2 margin at the well-differentiated component was not associated with local recurrence, association of marginal resection with local recurrence in their study suggests that wide margin should be secured throughout the surgery. However Morri’s study analyzed each case as either having a wide or marginal resection margin, without distinguishing resection margins at the dedifferentiated and the well-differentiated components. We note that, while wide resection margin is the standard surgical approach for DDL-E, further investigation into moderated wide resection margin at the well-differentiated component is warranted.

#### Limitations

The multi-centered and retrospective nature of this study is its main limitation. Variation in treatment strategies may exist across the 5 participated institutions. Recurrent and synchronous metastastic cases were excluded to reduce the effect of such variation. Although the protocols for adjuvant treatments varied by institutions, they shared a general strategy. Chemotherapy and radiotherapy were mostly performed adjuvantly. No institution performed neoadjuvant chemotherapy and neoadjuvant radiotherapy was performed in 2 of 107 cases. The study population consists entirely of Korean patients, which limits the generalizability of our findings to other ethnic groups. Future research involving more diverse populations is necessary to enhance the generalizability of these findings across different ethnicities.

## Conclusion

This is the largest restrospective cohort study investigating dedifferentiated liposarcoma of the extremities to-date. Localized DDL-E demonstrates low potential for metastasis and an excellent prognosis. Considering the high frequency of other primary malignancy and extrapulmonary metastasis in DDL-E, close monitoring of the patients including the sites other than the initial location of the DDL-E is strongly recommended. While a wide resection margin is the standard surgical approach for DDL-E, further investigation into moderated wide resection margin at the well-differentiated component is warranted.

## Data Availability

The data used and/or analyzed in this study are available from the corresponding author on request..

## Abbreviations

CI: Confidence Interval
DDL: Dedifferentiated liposarcoma
DDL-E: Dedifferentiated liposarcoma of the extremities
DDL-RP: Dedifferentiated liposarcoma of the RP
DSS: Disease specific survival
FISH: Fluorescence in situ hybridization
FNCLCC: Fédération Nationale des Centres de Lutte Contre le Cancer
Gy: Grey
HR: Hazard Ratio
LRFS: Local recurrence free survival
MDM2: Mouse Double Minute 2
MFS: Metastasis-free survival
MRI: Magnetic Resonance Imaging
ROC: Receiver Operating Characteristic
RP: Retroperitoneum
WDL: Well-differentiated Liposarcoma
WHO: World Health Organization
